# Supplementary material for: Seasonal dispersal and longitudinal migration in the Relict Gull Larus relictus across the Inner-Mongolian Plateau
Source: PeerJ. 2017 May 25;5:e3380. doi: 10.7717/peerj.3380 (PMC5446770; doi:10.7717/peerj.3380)
Supplement: Table S1 — By checking water level change since 1980s (Wang & Dou, 1998 ∗), we roughly ranked lake shrinkage as (A) no shrinkage or slight shrinkage (+), water surface area decreased by less than 10%; (B) medium (+ +), water surface area decreased by 10%∼50%; (C) serious (+ + +), water surface area decreased by more than 50%; or (D) dried up (+ + + +). ∗ Wang S, Dou H. 1998. Lakes of China. Beijing: Science Press. [file peerj-05-3380-s001.docx]

**Table S1: Human disturbance and habitat condition at major stopover sites for Relict Gull.**

By checking water level change since 1980S (Wang & Dou, 1998^*^), we roughly ranked lake shrinkage as (a) no shrinkage or slight shrinkage (+), water surface area decreased by less than 10%; (b) medium (++), water surface area decreased by 10%~50%; (c) serious (+++), water surface area decreased by more than 50%; or (d) dried up (++++).

^*^ Wang S, Dou H. 1998. *Lakes of China*. Beijing: Science Press

| **Stopover sites** | **Human Disturbance** | | | | | **Lake Shrinkage** |
| --- | --- | --- | --- | --- | --- | --- |
|  | **Tourism** | **Grazing** | **Fishing** | **Pollution** | **Dredging** |  |
| Chahai Nur |  |  | √ |  |  | +++ |
| Xiyan Nur |  |  |  |  |  | +++ |
| Anguli Nur | √ |  |  |  |  | ++++ |
| Cetian Reservoir | √ | √ |  |  |  | + |
| Huanggai Nur  &Sangai Nur | √ | √ |  | √ |  | ++++ |
| Wulanhushaohaizi & Baiyin Nur | √ | √ |  |  |  | ++ |
| Hanhaizi |  |  |  | √ |  | +++ |
| Huangqihai | √ | √ |  |  |  | ++ |
| Daihai | √ | √ | √ |  |  | ++ |
| Yanghe Reservoir |  |  | √ |  | √ | + |
