# Supplementary material for: Seasonal dispersal and longitudinal migration in the Relict Gull Larus relictus across the Inner-Mongolian Plateau
Source: PeerJ. 2017 May 25;5:e3380. doi: 10.7717/peerj.3380 (PMC5446770; doi:10.7717/peerj.3380)
Supplement: Table S2 — Threats include (A) intertidal mudflat reclamation; (B) tourism; (C) collection of sea food; (D) mining; (E) wind power generation; and (F) no threats. According to the spatial proportion affected, scope of threats was rated as (A) regional, <5%; (B) scattered, 5–15%; (C) widespread, 15–50%; or (D) entire, >50%. Durability of threats was rated as (A) short-term, <5 years; (B) medium-term, 5–20 years; (C) long-term, 20–100 years; or (D) permanent, >100 years. Location sees Fig. S1. [file peerj-05-3380-s002.docx]

**Table S2: Threats and their scope and duration in wintering locations for Relict Gulls on Bohai Bay.**

Threats include (a) intertidal mudflat reclamation; (b) tourism; (c) collection of sea food; (d) mining; (e) wind power generation; and (f) no threats.

According to the spatial proportion affected, scope of threats was rated as (a) regional, <5%; (b) scattered, 5-15%; (c) widespread, 15-50%; or (d) entire, >50%.

Durability of threats was rated as (a) short-term, <5 years; (b) medium-term, 5-20 years; (c) long-term, 20-100 years; or (d) permanent, >100 years.

Location sees Fig. S1.

| **Location** | **Coordinates** | **Threats** | **Scope** | **Durability** |
| --- | --- | --- | --- | --- |
| 1 | 39.4246°N,119.2945°E | a | d | c |
| 2 | 39.17457°N,118.8607°E | a | b | c |
| 3 | 39.11562°N,118.839°E | b | b | c |
| 4 | 39.13988°N,118.7359°E | c | b | a |
| 5 | 39.15545°N,118.678°E | a | d | d |
| 6 | 39.15036°N,118.6035°E | a | d | d |
| 7 | 39.1366°N,118.5536°E | a | d | d |
| 8 | 39.0275°N,118.3453°E | a | d | d |
| 9 | 39.0452°N,118.3084°E | c | b | a |
| 10 | 39.0400°N,118.2744°E | d | c | c |
| 11 | 39.1024°N,118.1921°E | d | b | a |
| 12 | 39.1887°N,118.1291°E | a | d | d |
| 13 | 39.2222°N,118.013°E | e | a | c |
| 14 | 39.2149°N,117.9638°E | a | a | c |
| 15 | 39.2085°N,117.9331°E | a | c | d |
| 16 | 39.1912°N,117.8681°E | a | c | d |
| 17 | 39.1762°N,117.8348°E | a | c | d |
| 18 | 39.1422°N,117.7958°E | a | c | d |
| 19 | 38.8428°N,117.6264°E | b | b | a |
| 20 | 38.7583°N,117.6066°E | a | c | d |
| 21 | 38.6169°N,117.5943°E | a | d | d |
| 22 | 38.5950°N,117.5993°E | a | d | d |
| 23 | 38.5673°N,117.6091°E | a | d | d |
| 24 | 38.5303°N,117.6268°E | a | d | d |
| 25 | 38.4901°N,117.6340°E | a | c | d |
| 26 | 38.4619°N,117.6641°E | a | d | d |
| 27 | 38.4297°N,117.6850°E | a | d | d |
| 28 | 38.4133°N,117.6957°E | a | d | d |
| 29 | 38.3908°N,117.7074°E | a | d | d |
| 30 | 38.3790°N,117.7043°E | a | d | d |
| 31 | 38.3263°N,117.7657°E | d | a | a |
| 32 | 38.3126°N,117.8449°E | a | d | d |
| 33 | 38.2652°N,117.8473°E | b | a | a |
| 34 | 38.2483°N,117.8781°E | f |  |  |
| 35 | 38.2068°N,117.9685°E | f |  |  |
| 36 | 38.1927°N,118.0754°E | a | d | d |
| 37 | 38.1218°N,118.0361°E | a | b | c |
