# Supplementary material for: Seasonal dispersal and longitudinal migration in the Relict Gull Larus relictus across the Inner-Mongolian Plateau
Source: PeerJ. 2017 May 25;5:e3380. doi: 10.7717/peerj.3380 (PMC5446770; doi:10.7717/peerj.3380)
Supplement: Table S3 [file peerj-05-3380-s003.docx]

**Table S3: Performance of satellite transmitters for Relict Gulls breeding at Hongjian Nur in 2007, 2008 and 2010.**

| **Individuals** | **Transmitting Cycle (h on / h off)** | **Tracking Period** | **Tracking Days** | **Total Locations** | **Location Class** | | | | | | |
| --- | --- | --- | --- | --- | --- | --- | --- | --- | --- | --- | --- |
|  |  |  |  |  | **3** | **2** | **1** | **0** | **A** | **B** | **Z** |
| G1 | 10 / 47 | 2007.07.13-2007.09.09 | 59 | 23 | 1 | 2 | 0 | 1 | 12 | 7 | 0 |
| G2 | 10 / 47 | 2007.08.01-2007.09.04 | 35 | 8 | 0 | 0 | 0 | 1 | 2 | 5 | 0 |
| G3 | 8 / 15 | 2008.06.25-2009.11.30 | 524 | 942 | 73 | 137 | 163 | 128 | 224 | 206 | 11 |
| G4 | 8 / 15 | 2008.06.25-2012.07.05 | 1471 | 2128 | 155 | 330 | 483 | 339 | 396 | 389 | 36 |
| G5 | 8 / 15 | 2008.06.25-2013.04.26 | 1765 | 2937 | 183 | 414 | 567 | 544 | 579 | 606 | 44 |
| G6 | 8 / 15 | 2008.06.25-2008.10.13 | 111 | 345 | 20 | 42 | 65 | 61 | 78 | 73 | 6 |
| G7 | 8 / 15 | 2008.06.25-2010.04.15 | 660 | 925 | 59 | 121 | 144 | 130 | 239 | 220 | 12 |
| G8 | 10 / 47 | 2010.07.01-2011.01.04 | 127 | 121 | 17 | 33 | 27 | 14 | 22 | 8 | 0 |
| G9 | 8 / 23 | 2010.07.02-2011.03.03 | 245 | 232 | 15 | 43 | 26 | 15 | 60 | 70 | 3 |
| G10 | 8 / 23 | 2010.07.01-2010.09.17 | 79 | 176 | 6 | 17 | 28 | 24 | 45 | 51 | 5 |
| G11 | 8 / 23 | 2010.07.01-2012.09.01 | 793 | 1345 | 98 | 215 | 255 | 150 | 261 | 338 | 28 |
