# Supplementary material for: Seasonal dispersal and longitudinal migration in the Relict Gull Larus relictus across the Inner-Mongolian Plateau
Source: PeerJ. 2017 May 25;5:e3380. doi: 10.7717/peerj.3380 (PMC5446770; doi:10.7717/peerj.3380)
Supplement: Table S4 — All of the recorded gulls were banded when they were chicks. The recovery records indicate that juveniles distribute in a larger wintering range relative to satellite tracked adults. Data were accessed from China National Bird Banding Database ( http://www.chinanbbc.net). [file peerj-05-3380-s004.doc]

**Table S4: Recovery records of juvenile Relict Gulls (≤two-year-old)** **in non-breeding season.**

All of the recorded gulls were banded when they were chicks. The recovery records indicate that juveniles distribute in a larger wintering range relative to satellite tracked adults. Data were accessed from China National Bird Banding Database ([http://www.chinanbbc.net](http://www.chinanbbc.net/)).

| **Metal ring code** | **Legflag code** | **Banding site** | **Banding coordinate** | **Banding date** | **Recovery site** | **Recovery coordinate** | **Recovery date** |
| --- | --- | --- | --- | --- | --- | --- | --- |
| M-264059 | — | Alakol Lake, Kazakhstan | 46°6.0′N, 81°50.0′E | 1975-6-21 | Huimin, China | 37°30.0′N, 117°30.0′E | 1977-2-12 |
| M-352309 | — | Alakol Lake, Kazakhstan | 46°6.0′N, 81°50.0′E | 1977-6-24 | Yimen, China | 24°40.0′N, 102°10.0′E | 1978-8-1 |
| I08-8073^*^ | — | Hongjian Nur, China | 39°8.3′N, 109°52.2′E | 2007-6-26 | Dongtai, China | 32°47.0′N, 120°21.0′E | 2008-6-2 |
| I03-3028 | 15 | Hongjian Nur, China | 39°8.3′N, 109°52.2′E | 2008-6-25 | Luannan, China | 39°3.80′N, 118°13.1′E | 2009-5-25 |
| I03-3141 | C0 | Hongjian Nur, China | 39°8.3′N, 109°52.2′E | 2008-6-25 | Luannan, China | 39°3.80′N, 118°13.1′E | 2009-5-25 |
| I03-3143 | C2 | Hongjian Nur, China | 39°8.3′N, 109°52.2′E | 2008-6-25 | Luannan, China | 39°3.80′N, 118°13.1′E | 2009-5-25 |
| I03-3009 | 25 | Hongjian Nur, China | 39°8.3′N, 109°52.2′E | 2008-6-25 | Luannan, China | 39°3.80′N, 118°13.1′E | 2009-5-28 |
| C-810482 | — | Tandinskiy, Russia | 51°19.9′N, 94°31.1′E | 2008-6-27 | Jinzhou, China | 39°6.1′N, 121°41.2′E | 2008-11-20 |

^*^ This bird was found died
