# Supplementary figures and images for: Seasonal dispersal and longitudinal migration in the Relict Gull Larus relictus across the Inner-Mongolian Plateau
Source: PeerJ. 2017 May 25;5:e3380. doi: 10.7717/peerj.3380 (PMC5446770; doi:10.7717/peerj.3380)

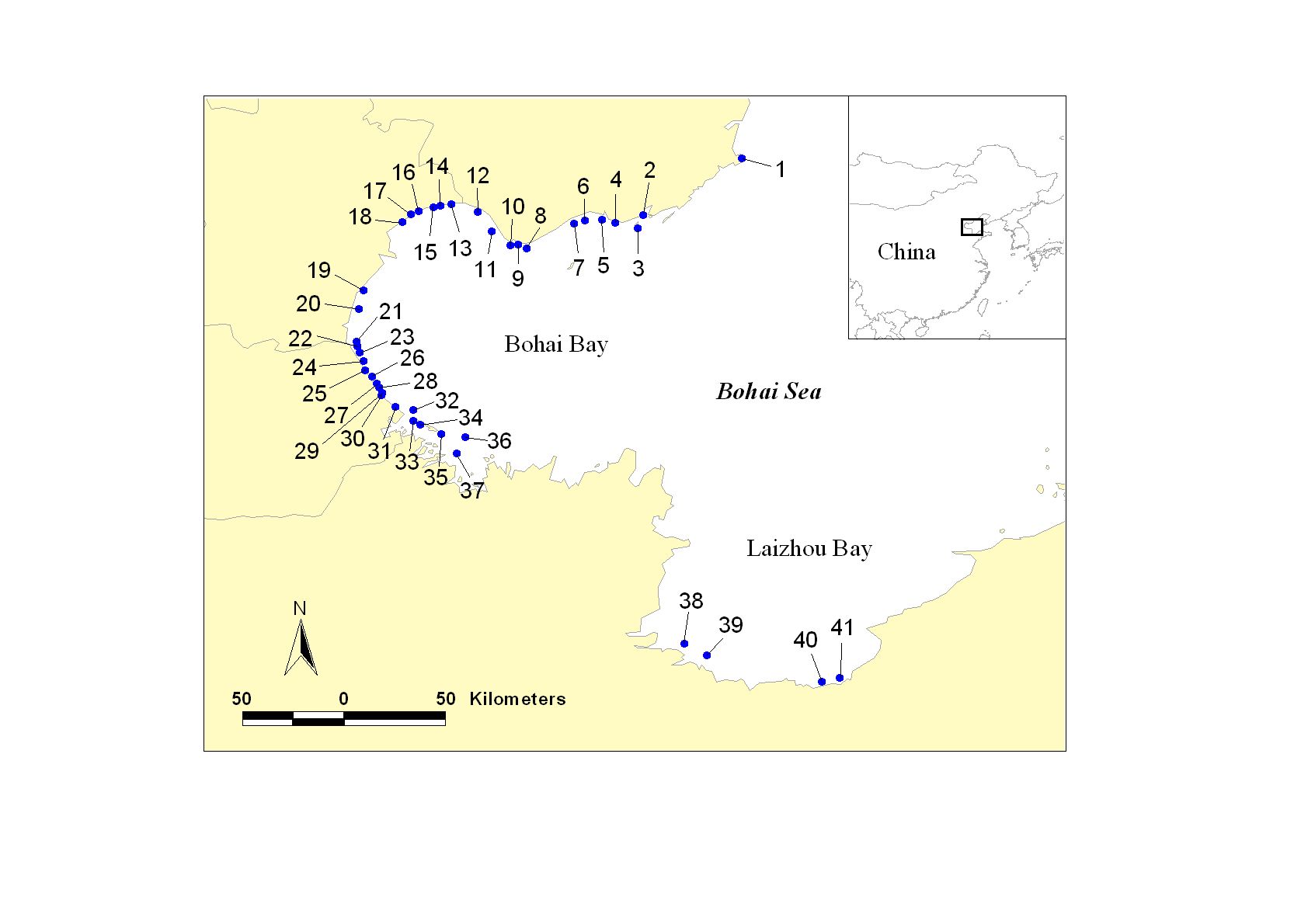

Supplement: Figure S1 — 1-Luanhe River Estuary, Leting Co.; 2-Daqinghe River Estuary, Leting Co.; 3-Puti Island, Leting Co.; 4-Xidajian, Leting Co.; 5-Xiaoqinghe River Estuary, Leting Co.; 6-Shuohe River Estuary, Tanghai Co.; 7-Qinglonghe River Estuary, Tanghai Co.; 8-Zuidong, Luannan Co.; 9-Nanbao, Luannan Co.; 10-Nanbao Oil Field, Luannan Co.; 11-Beibao, Luannan Co.; 12-Heiyanzi, Fengnan Co.; 13-Dashentang Power Field, Hangu of Tianjin; 14-Dashentang Wharf, Hangu of Tianjin; 15-Dongjiang Power Field, Hangu of Tianjin; 16-Central Fishing Port, Hangu of Tianjin; 17-Caijiabao, Hangu of Tianjin; 18-Chengtougu, Tanggu of Tianjin; 19-Haibinyuchang, Tanggu of Tianjin; 20-Duliujian River Estuary, Tianjin; 21-Ziyaxinhe River Estuary, Tianjin; 22-Qikou, Huanghua; 23-Zhangjuhe River, Huanghua; 24-Houtangbao, Huanghua; 25-Nanpaihe River, Huanghua; 26-Fanjiabao, Huanghua; 27-Guanjiabao, Huanghua; 28-Xiaoxinbao, Huanghua; 29-Xujiabao, Huanghua; 30-Yangjiabao, Huanghua; 31-Fengjiabao, Huanghua Harbor; 32-East Beach of Zhongtiegongsi, Huanghua Harbor; 33-Zhangweixinhe River Estuary, Huanghua Harbor; 34-East Bay of Dahekou Island, Huanghua Harbor; 35-Majiahe River Estuary, Wudi Co.; 36-Binzhou Harbor; 37-Taoerhe River Estuary, Wudi Co.; 38-Zhimaihe River Estuary, Guangrao Co.; 39-Xiaoqinghe River Estuary, Shouguang Co.; 40-Weihe River Estuary, Changyi; 41-Jiaolaihe River Estuary, Changyi [file peerj-05-3380-s005.jpg]

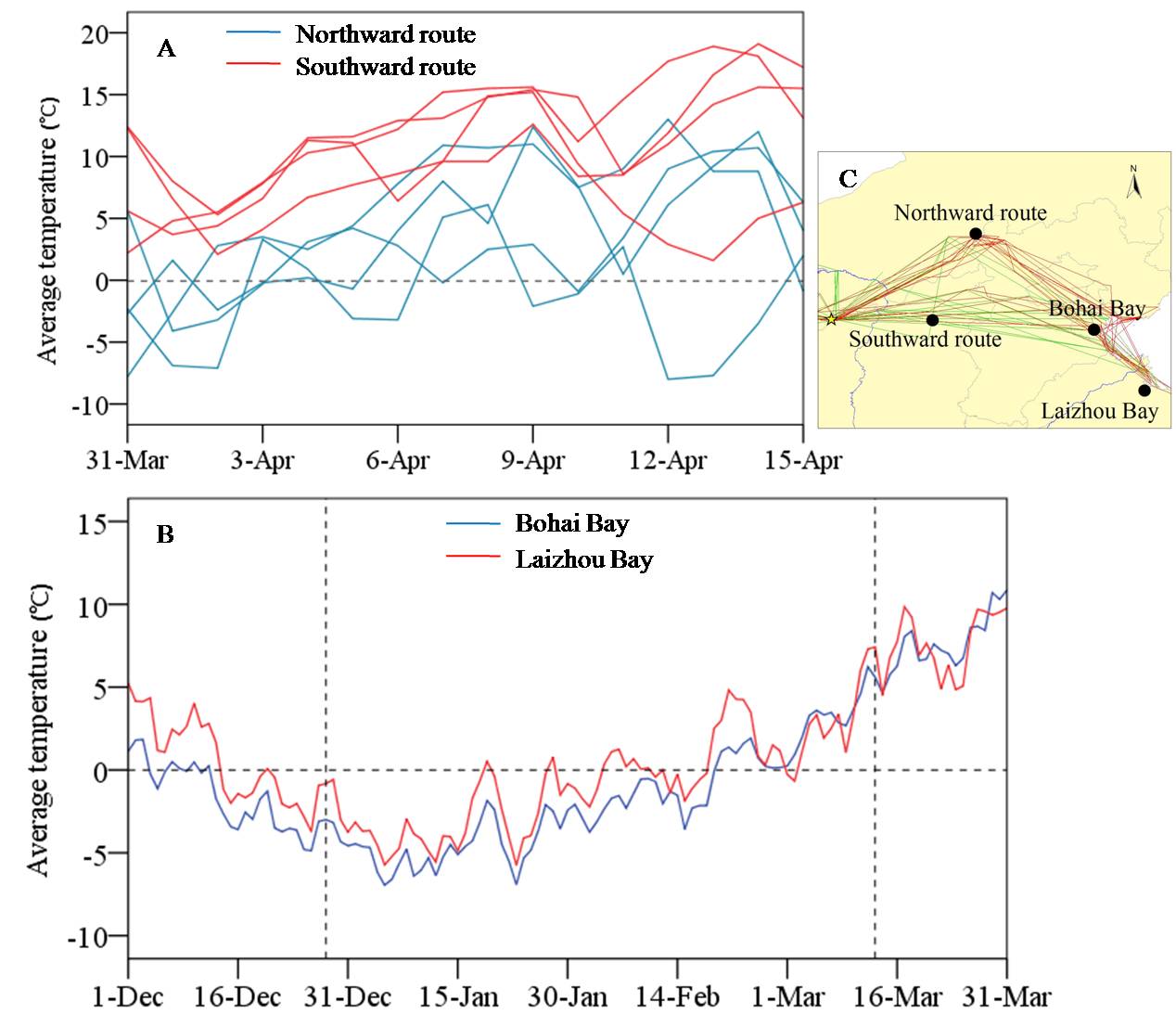

Supplement: Figure S2 — (A) Temperature difference between southward and northward routes during spring migration from 2009 to 2012. Daily temperature varied greatly (range = −8 13;) in northward route among years, which may have impact on the onset and arrival date of spring migration. There are considerable proportion of days with temperature below zero in northward routes, which may explain why Relict Gulls tended to follow warmer southward routes in spring migration. (B) Difference of temperature (average from 2008 to 2012) between Bohai and Laizhou Bay during winter dispersal. The two reference lines for X axis indicate average time period (28 Dec–13 Mar) for Relict Gulls to disperse from frozen Bohai Bay to warmer Laizhou Bay. Temperature data were extracted from four typical meteorological station s (C) to represent northward & southward routes and Bohai & Laizhou Bay, respectively. Data accessed from NOAA (available at https://gis.ncdc.noaa.gov/maps/ncei#app=clim&cfg=cdo&theme=hourly&layers=1&node=gis). [file peerj-05-3380-s006.jpg]
